# Risk Factors for Malaria Infection and Seropositivity in the Elimination Area of Grand’Anse, Haiti: A Case–Control Study among Febrile Individuals Seeking Treatment at Public Health Facilities

**DOI:** 10.4269/ajtmh.20-0097

**Published:** 2020-05-26

**Authors:** Ruth A. Ashton, Vena Joseph, Lotus L. van den Hoogen, Kevin K. A. Tetteh, Gillian Stresman, Matt Worges, Thomas Druetz, Michelle A. Chang, Eric Rogier, Jean Frantz Lemoine, Chris Drakeley, Thomas P. Eisele

**Affiliations:** 1 Center for Applied Malaria Research and Evaluation, Tulane School of Public Health and Tropical Medicine, New Orleans, Louisiana;; 2 Department of Infection Biology, London School of Hygiene and Tropical Medicine, London, United Kingdom;; 3 Department of Social and Preventive Medicine, University of Montreal School of Public Health, Montreal, Canada;; 4 Division of Parasitic Diseases and Malaria, Malaria Branch, Centers for Disease Control and Prevention, Atlanta, Georgia;; 5 Programme National de Contrôle de la Malaria, Ministry of Public Health and Population, Port-au-Prince, Haiti

## Abstract

The island of Hispaniola aims to eliminate malaria by 2025; however, there are limited data to describe epidemiologic risk factors for malaria in this setting. A prospective case–control study was conducted at four health facilities in southwest Haiti, aiming to describe factors influencing the risk of current and past malaria infection. Cases were defined as individuals attending facilities with current or recent fever and positive malaria rapid diagnostic test (RDT), while controls were those with current or recent fever and RDT negative. Serological markers of recent and cumulative exposure to *Plasmodium* were assessed using the multiplex bead assay from dried blood spots and used for alternate case definitions. Kuldorff’s spatial scan statistic was used to identify local clusters of infection or exposure. Logistic regression models were used to assess potential risk factors for RDT positivity and recent exposure markers, including age-group, gender, and recruiting health facility as group-matching variables. A total of 192 cases (RDT positive) and 915 controls (RDT negative) were recruited. Consistent spatial clusters were identified for all three infection and exposure metrics, indicating temporal stability of malaria transmission at these sites. Risk factors included remoteness from health facilities and household construction, furthermore, insecticide-treated net ownership or use was associated with reduced odds of RDT positivity. These findings indicate the malaria risk in Grand’Anse is driven primarily by location. Travel, occupation, and other behavioral factors were not associated with malaria. These data can support the National Malaria Program to refine and target their intervention approaches, and to move toward elimination.

## INTRODUCTION

**Figure 1.**
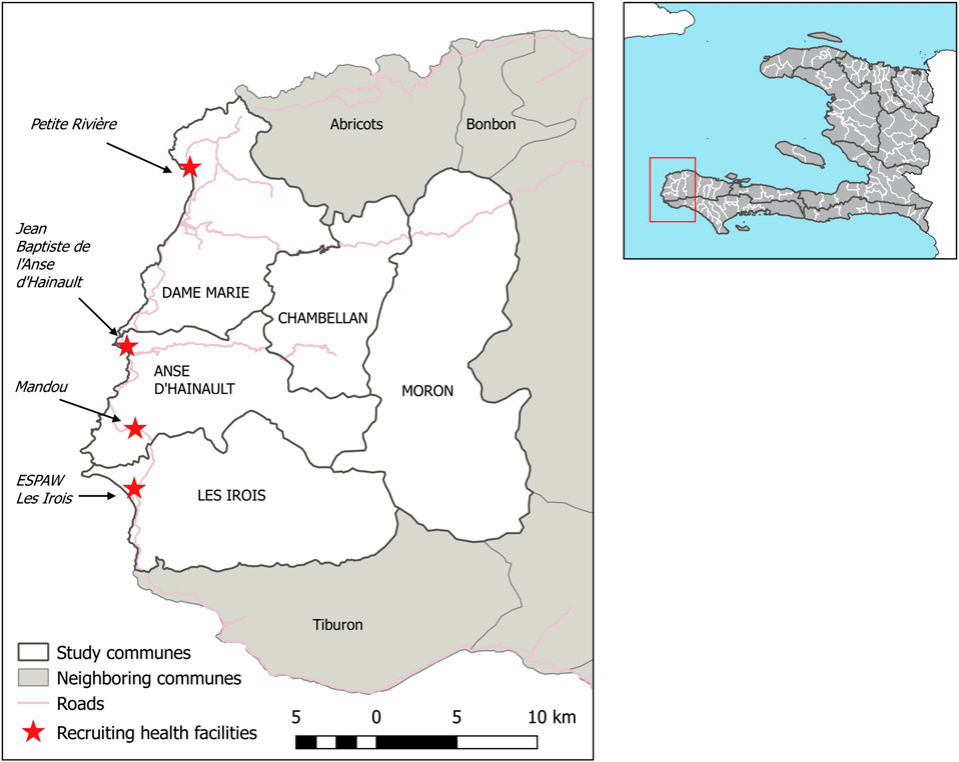
Map of the five study communes (white fill) and neighboring communes (gray fill), with the four health facilities recruiting participants to the study indicated by red markers. The location of the study area within Haiti is shown by the red box in the locator map, with department boundaries (gray line) and commune boundaries (white line).

The island of Hispaniola is the only location in the Caribbean with ongoing malaria transmission, and most malaria cases in Hispaniola occur in Haiti.^1^ Haiti and the Dominican Republic are targeting malaria elimination by 2025. Haiti is using a multipronged approach including improved surveillance systems, vector control, expansion of malaria case management to the community level, and piloting geographically targeted interventions such as mass drug administration. The Grand’Anse department in southwest Haiti (Figure 1) experiences approximately one-third to half of all malaria cases reported in Haiti and is the focus of many of the targeted interventions.

Limited data are available from Haiti to describe population groups or characteristics which are associated with the increased risk of malaria. To help Haiti achieve malaria elimination, data describing demographic, behavioral, or geographic risk factors are needed by the National Malaria Control Program (Programme National de Contrôle de la Malaria [PNCM]) to assist with refining and targeting intervention and elimination approaches. Formative research suggests that populations in malaria-risk areas of Haiti associate malaria with “dirty environments” (swamps, trash and dirty yards, and proximity to livestock) but believe that there are no clearly defined high-risk populations because “mosquitoes are everywhere” and are perceived to bite people indiscriminately.^2^ *Anopheles albimanus* is the principal malaria vector in Haiti, and although they are generally understood to bite outdoors more than indoors, data on the vector behavior in Haiti are inconclusive and limited.^3^ There is currently no evidence of insecticide resistance in *An. albimanus* in Haiti.

Case–control studies are particularly suited to generating evidence of risk factors for rare diseases and have been used for malaria risk factor assessments in settings as varied as Ethiopia,^4,5^ Namibia,^6^ China,^7^ and Indonesia.^8^ A prior case–control study conducted in Haiti during 2012–2014 found no evidence for a protective effect of consistent insecticide-treated net (ITN) use against symptomatic malaria following a national ITN distribution, but in a context of low rates of consistent ITN use (13% reported using an ITN on all 14 nights in the 2 weeks before the onset of illness).^9^ The 2012–2014 case–control study identified rudimentary roofing material as a risk factor for malaria and found some protective effect from the use of indoor (non-residual) pyrethroid-based insecticide spray.

Insecticide-treated nets remain a key vector control intervention targeted to the high-risk population in Haiti, primarily funded through the Global Fund. The PNCM last implemented a targeted ITN distribution campaign in June and November 2017 to 33 communes considered most at risk of malaria, including all communes of the Grand’Anse department.^10^ Estimated post-distribution ITN coverage in the three communes included in the current study ranged from 62.8% to 69.7% by commune (Haiti PNCM, unpublished data).

The aim of this study was to describe the major factors influencing who is at increased risk of current malaria infection and recent exposure in five communes of the Grand’Anse department, including temporal, spatial, demographic, and behavioral factors, in addition to access to and use of common malaria interventions. These findings can support the PNCM to refine and appropriately target malaria elimination activities.

## METHODS

### Study area.

The Grand’Anse department is located in the far southwest of Haiti, a forested area with a population of less than half a million. Settlements are more densely concentrated along the coast and along the road network, with smaller villages located further inland in the mountains. Most roads are unsurfaced, with settlements away from the coast and within the mountains commonly accessible by foot or motorbike only. The primary seasonal peak of malaria is generally from November to January, with a smaller peak in transmission during June and July. Grand’Anse is the department with the highest malaria incidence in Haiti, estimated at 18.1 per 1,000 population in 2017, and accounted for 45% of all confirmed malaria cases nationally.^2^

The Malaria Zero Alliance was established with the aim to assist ministries of health with malaria elimination on Hispaniola. The five communes in Grand’Anse with the highest malaria burden (Anse d’Hainault, Chambellan, Dame Marie, Les Irois, and Moron) have been targeted to receive an aggressive package of malaria elimination strategies and are the focus of operational research activities for Malaria Zero. Sixteen public health facilities are operational in these five communes. Four health facilities were purposively selected for inclusion in the case–control study; the selected facilities had high patient flow and a high proportion of malaria positivity during an “easy access group” survey in 2017.^11^ The selected sites (Figure 1) were two rural health facilities (Petite Rivière and Mandou), a health center in a small town (Les Irois), and a hospital (Jean Baptiste de l’Anse d’Hainault). All four facilities were accessible by vehicle throughout the study period. Study teams were provided with contingency stocks of malaria rapid diagnostic tests (RDTs), chloroquine, and primaquine to prevent any temporary stock-outs during the study period.

### Study design.

A prospective case–control study was conducted at the four study health facilities from April to August 2018. The primary definition of a case was an individual attending a participating health facility with febrile illness who had a positive RDT result, whereas controls were individuals attending the participating health facilities with febrile illness who were confirmed to be negative for malaria by RDT. Two additional case/control definitions were used 1) presence/absence of serological markers of recent exposure to *Plasmodium falciparum* and 2) presence/absence of serological markers of cumulative exposure to *P. falciparum*. The exclusion criteria for all case and control definitions were younger than 6 months, any severe disease, taking an antimalarial drug in the 14 days before visiting the health facility, and residence outside the commune of the recruiting health facility. All eligible individuals with a positive RDT were invited to participate in the study (primary case definition). Each facility had a recruitment ceiling for the number of controls by age- and gender group for each month, based on the proportion of malaria cases in each category in the most recently available (2016) surveillance data.

A target sample size of 605 cases and 1,210 controls was required to detect an odds ratio of 2.0 (based on 5% prevalence of exposure of interest in controls and design effect of 1.5), with 80% power and a significance level of 5%. The study duration was set at 16 weeks, and data collection was timed to capture approximately 8 weeks of low-transmission season (April–May) and 8 weeks of increased transmission (June–July).

### Data collection.

Individuals receiving a malaria RDT as part of their consultation at the four participating health facilities were referred by clinicians to the study team as potential study participants. The RDTs used at health facilities were First Response HRP2 (I13FRC30, Premier Medical Corporation, Nani Daman, India) or SD Bioline HRP2 (05FK50, Standard Diagnostics, Yongin-si, Republic of Korea). Consenting participants had their RDT result recorded and were requested to provide an additional finger-prick blood sample at the time of recruitment for the preparation of dried blood spots (DBS) on filter paper for subsequent serological analysis. Basic demographic information and location of residence were collected at the facility by the study team to enable a follow-up visit to the household, with an appointment scheduled if necessary. During the household follow-up visit, a questionnaire was used to collect basic demographic information about all household residents and more detailed information about the case/control individual. The questionnaire included the education level and occupation of the household head, asset ownership, livestock ownership, and where livestock are usually kept overnight. Household construction, and presence and brand(s) of mosquito nets were observed by the interviewer at the household. The questionnaire also collected information on all household residents regarding history of fever in last 2 weeks, treatment-seeking behavior, and any treatment received. Questions only asked to the case/control included travel history, occupation, usual sleep/wake times, and times they typically go inside for the night and leave the house in the morning. Malaria incidence in 2017 was extracted from routine surveillance data for each reported travel destination commune. Entomological data were collected at a subset of households and will be reported in a separate publication (Joseph et al., in preparation).

### Data processing.

Questionnaire data were collected electronically on Android tablets using CommCare software (Dimagi, Cambridge, MA), and all household global positioning system (GPS) coordinates were recorded using the tablet. Questionnaire data were automatically pushed to a secure cloud-based server using a local mobile phone network connection. Data were extracted from the CommCare server for cleaning and analysis using Stata 14.0 (StataCorp, College Station, TX). Maps were created using QGIS 3.6.

Household wealth was classified according to the ownership of various household assets using principal components analysis (PCA).^12^ A binary variable to differentiate nonpeak and peak malaria seasons was generated by qualitatively examining the ratio of cases-to-controls recruited each week. The peak season was defined as June 4, 2018 onward, when cases first exceeded 20% of weekly participant recruitment.

Raster data collected included geostatistically derived estimates of accessibility (time to travel to the nearest city of > 50,000 inhabitants, 1 km resolution), access to health services (walking time to the nearest health facility, 1 km resolution), elevation at 1 arc second (approximately 30 m) resolution,^13^ land cover from the International Geosphere-Biosphere Programme at 1 km resolution,^14^ total monthly rainfall from the climate hazards infrared precipitation with stations (CHIRPS) database at 5 km resolution,^15^ and monthly mean normalized difference vegetation index from the moderate-resolution imaging spectroradiometer (MODIS) at 1 km resolution.^16^ Predicted underlying transmission intensity (modeled malaria incidence 2014–2017, 300 m resolution) was generated by the Malaria Atlas Project (Battle et al., in preparation) as part of the wider Malaria Zero project. Briefly, the modeled malaria incidence surface was generated using monthly health facility data from 2014 to 2017, case-tracing data, serological data from easy access group surveys and transmission assessment surveys, treatment-seeking probability based on distance to the nearest health facility, environmental covariates (e.g., aridity, distance to water, elevation, temperature, and vegetation index), and denominator population estimated from Facebook high-resolution population maps. Data from all spatial layers were extracted to participants’ household location.

For all participants who provided DBS, serum antibody levels to a panel of 19 *Plasmodium* antigens were determined using the multiplex bead assay (MBA) at the National Public Health Laboratory (Laboratoire National de la Santé Publique [LNSP]) in Port-au-Prince.^17–19^ Briefly, antigens were covalently coupled to unique bead regions as previously described,^20^ and then processed for multiplex antibody (IgG) detection using a one-step protocol.^18^ Median fluorescence intensity (MFI) was recorded for each sample and corrected for background reactivity, and then log-transformed.^17^ A finite mixture model was used to define positivity, assuming two normal distributions with 3 SD threshold calculations.^21^ A binary metric describing the presence of malaria antibodies representing recent exposure was defined as positivity to one antigen target (Etramp 5 Ag 1), whereas an indicator of longer term malaria exposure was defined as positivity against at least one of two antigens (PfAMA1 and PfMSP1-19).^19^ These two binary metrics were used to define additional case and control definitions for recent and cumulative exposure to *P. falciparum.* Serology data from children younger than 1 year were not retained because of potential confounding from maternal antibodies.^22^

### Data analysis.

To identify local clusters of cases, Kuldorff’s spatial scan statistic was used with SaTScan version 9.6.^23^ Briefly, a spatial-only Bernoulli model was used whereby an elliptical window of variable size is scanned over case and control location data to identify areas with a higher than expected proportion of cases, against a null hypothesis of no clustering. The Limiting criteria of ≥ 2 cases and < 50% of the study population (cases and controls) were used to define a cluster, allowing identification of a wide range of cluster sizes. The date of recruitment was not considered in the spatial scan statistic, and only clusters with *P* < 0.05 were considered significant. Gini coefficients were used to determine the best combination of nonoverlapping clusters.^24^ Spatial scan statistics were generated for each case/control definition: 1) by RDT result, 2) seropositivity against markers of recent *P. falciparum* exposure, and 3) seropositivity against markers of cumulative *P. falciparum* exposure. For each cluster, the reported relative risk (RR) is defined as the estimated risk within the cluster divided by the estimated risk outside the cluster, with cluster *P*-values based on 999 Monte Carlo simulations. Analysis was repeated for facility-specific datasets, yielding similar results to analysis of the entire recruited study population.

Potential malaria risk factors were assessed by logistic regression models in Stata 14.0 (Stata Corp., College Station, TX) for two different outcomes: 1) RDT positive and 2) presence of markers of recent exposure to *P. falciparum*. Cases and controls were not individually matched, but all logistic regression models included age-group (< 5, 5–14, 15–29, 30–45, and > 45 years), gender, and the recruiting health facility as group-matching variables. The transmission season (peak or off-peak) was preselected for inclusion in RDT models only, whereas predicted underlying transmission intensity was included in models for both outcomes. Covariates tested in models included household construction, ownership and the use of personal protection methods (e.g., ITNs), household wealth, livestock ownership and overnight proximity to the household, and accessibility of household to health facilities and urban areas. Models included the predicted malaria incidence at the household location to adjust for variation in transmission intensity and allow models to focus on demographic and behavioral risk factors. Covariates tested in the recent exposure model were limited to those variables expected to be stable over a period of weeks to months, for example, household construction, accessibility, net ownership, and livestock ownership. Models were not generated for cumulative markers of *P. falciparum* infection because of challenges in defining and collecting data on historical risk factors, particularly resulting from expected changes in livelihood and housing following the passage of Hurricane Matthew in October 2016.

### Ethical considerations.

The study protocol was approved by the National Bioethics Committee of the Haitian Ministry of Public Health and Population (1718-20), Tulane University (2017-366), and the London School of Hygiene and Tropical Medicine (14556). The study protocol and institutional ethics determinations were reviewed and approved by the U.S. CDC and Office of the Associate Director of Science at the Center for Global Health, and CDC investigators were determined not to be engaged in human subject research.

Consent from all participants was recorded electronically on tablets during recruitment at health facilities and confirmed at the follow-up interview. Consent for individuals younger than 18 years was given by a parent or guardian. Mature minors (aged 16–17 years and either pregnant, a parent, or head of household) were able to provide consent directly. All individuals with a positive RDT result received the national first-line malaria treatment (chloroquine and a single dose of primaquine) during their consultation at the health facility.

## RESULTS

### Descriptive analysis.

A total of 192 cases (RDT positive) and 915 controls (RDT negative) were recruited into the study and completed a follow-up interview, comprising only one-third of the target case sample size (enrollment flowchart is shown in Supplemental Figure 1). Logistical constraints prevented extension of the data collection period. All health facilities in Haiti experienced a drop in confirmed malaria cases in 2018. During the follow-up interview, errors in data collection led to information on ITN use not being recorded for 146 individuals (32 RDT positive and 114 RDT negative). Missing ITN use data were more common among under-fives (33% of missing versus 21% of non-missing), male-headed households, and individuals recruited at Mandou facility (56% of missing). Multivariate models were consequently restricted to the 961 (87%) individuals with full data, but all other results use the full dataset (*N* = 1,107). The available sample size (after exclusion of those with missing ITN use data) permitted detection of an odds ratio (OR) of 2.0 based on 20% prevalence of exposure among controls, with 80% power, design effect of 1.5, and alpha 5%; or detection of an OR of 2.5 with 9% prevalence of exposure among controls.

**Figure 2.**
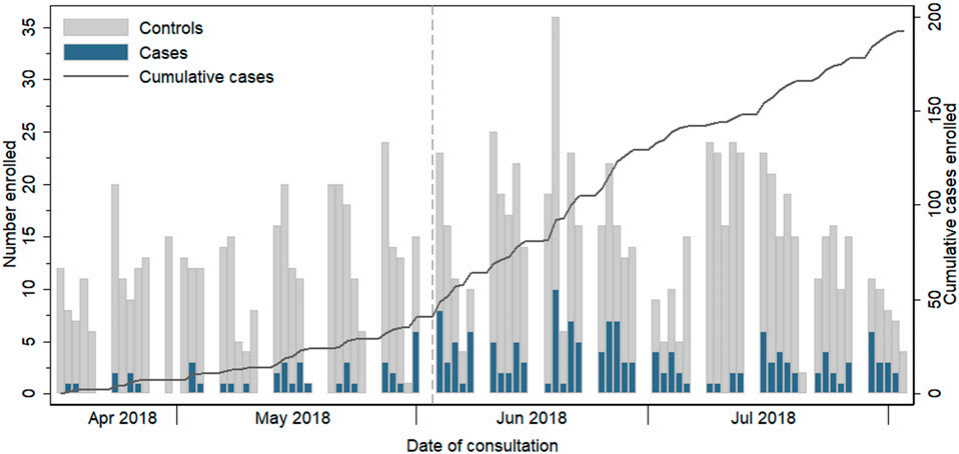
Stacked bar chart describing number of cases and controls (by rapid diagnostic test diagnosis) recruited at all four health facilities each day and cumulative case recruitment (secondary *y* axis). The transition from off-peak season (before fourth June) and peak transmission season (fourth June onward) is indicated by a vertical dashed line.

The enrolment of cases and controls (defined by RDT result) over time is shown in Figure 2. The median time between recruitment (day of malaria testing at health facility) and the follow-up interview at home was 1 day (range 0–43 days); 92% of participants were followed up at home within 7 days.

A summary of participant recruitment and key demographic characteristics is presented in Table 1, with breakdown using both RDT and recent exposure seropositivity case–control definitions. A description of participant demographics using markers of cumulative *P. falciparum* exposure as the case/control definition is shown in Supplemental Table 1. Of 1,055 individuals with serology data, 30.1% were found to have markers of recent exposure to *P. falciparum* (irrespective of RDT result). Of the 1,055 individuals with both RDT and serology results, 321 (30.4%) were negative by RDT, recent and cumulative markers of exposure. Comparing RDT positivity and markers of recent exposure, 158 (15.0%) had markers of recent exposure to *P. falciparum* but were RDT negative, whereas 28 (2.7%) were RDT positive but seronegative for recent exposure markers, and 159 (15.1%) were both RDT positive and had markers of recent exposure. A comprehensive assessment of univariate associations between case–control classification (by RDT and serological markers of recent exposure) and demographic, household, behavioral, and environmental variables is presented in Supplemental Table 2.

**Table 1:** Demographic characteristics of study participants and chi-squared *P*-values using two different case–control classifications: result of malaria RDT and presence of antibodies representing recent exposure (positivity to antigen target Etramp 5 Ag 1) in the multiplex bead-based assay

	RDT positivity			Recent exposure marker		
	Case, *n* (%)	Control, *n* (%)	*P-*value	Case, *n* (%)	Control, *n* (%)	*P-*value
Gender of case/control						
Male	97 (50.5)	417 (45.6)	0.211	143 (45.1)	344 (46.6)	0.654
Female	95 (49.5)	498 (54.4)		174 (54.9)	394 (53.4)	
Age-group (years)						
< 5	26 (13.5)	225 (24.6)	0.002	40 (12.6)	191 (25.9)	< 0.001
5–14	60 (31.3)	233 (25.5)		87 (27.4)	196 (26.6)	
15–29	64 (33.3)	219 (23.9)		90 (28.4)	181 (24.5)	
30–44	28 (14.6)	159 (17.4)		64 (20.2)	115 (15.6)	
45+	14 (7.3)	79 (8.6)		36 (11.4)	55 (7.5)	
Recruiting facility						
Les Irois	56 (29.2)	177 (19.3)	< 0.001	77 (24.3)	146 (19.8)	< 0.001
Mandou	42 (21.9)	174 (19.0)		76 (24.0)	130 (17.6)	
Jean Baptiste de l’Anse d’Hainault	15 (7.8)	282 (30.8)		52 (16.4)	245 (33.2)	
SKS Petite Rivière	79 (41.2)	282 (30.8)		112 (35.3)	217 (29.4)	
Time lived in the current community (years)						
< 1	3 (1.6)	18 (2.0)	0.722	4 (1.3)	13 (1.8)	0.512
1–2	30 (15.6)	140 (15.3)		43 (13.6)	126 (17.1)	
3–4	9 (4.7)	61 (6.7)		19 (6.0)	50 (6.8)	
5–9	8 (4.2)	58 (6.3)		20 (6.3)	46 (6.2)	
10 +	21 (10.9)	89 (9.7)		39 (12.3)	69 (9.4)	
Whole life (all ages)	121 (63.0)	549 (60.0)		192 (60.6)	434 (58.8)	
Any travel in previous 12 months						
Yes	44 (22.9)	200 (21.9)	0.748	67 (21.1)	170 (23.0)	0.498
No	148 (77.1)	715 (78.1)		250 (78.9)	568 (77.0)	
Livestock ownership						
Yes	145 (75.5)	572 (62.5)	0.001	226 (71.3)	447 (60.6)	0.001
No	47 (24.5)	343 (37.5)		91 (28.7)	291 (39.4)	
Roof material						
Thatch/palm leaf/bamboo	7 (3.7)	24 (2.6)	0.020	11 (3.5)	20 (2.7)	0.092
Canvas/tent	60 (31.3)	201 (22.0)		83 (26.2)	162 (22.0)	
Iron sheets	115 (59.9)	597 (65.3)		205 (64.7)	478 (64.8)	
Tiles/cement	9 (4.7)	89 (9.7)		17 (5.4)	74 (10.0)	
Other	1 (0.5)	4 (0.4)		1 (0.3)	4 (0.5)	
Wall material						
No walls or palm leaf/bamboo	52 (27.1)	143 (15.6)	0.003	71 (22.4)	113 (15.3)	0.013
Bamboo and mud or stone and mud	55 (28.7)	263 (28.7)		101 (31.9)	202 (27.4)	
Wood plank or salvaged wood	8 (3.7)	48 (5.3)		11 (3.5)	44 (6.0)	
Canvas or tent	5 (2.6)	61 (6.7)		17 (5.4)	49 (6.6)	
Metal sheet	2 (1.0)	13 (1.4)		4 (1.3)	10 (1.4)	
Cement block or stone and cement	71 (37.0)	387 (42.3)		113 (35.7)	320 (43.4)	
Occupation of case/control						
< 16 years	92 (47.9)	477 (52.1)	0.056	134 (42.3)	405 (54.9)	< 0.001
Student	35 (18.2)	105 (11.5)		46 (14.5)	85 (11.5)	
Agriculture or fishing	26 (13.5)	111 (12.1)		53 (16.7)	76 (10.3)	
Day labor	8 (4.2)	23 (2.5)		9 (2.8)	22 (3.0)	
Shopkeeper	22 (11.5)	130 (14.2)		59 (18.6)	89 (12.1)	
Other	9 (4.7)	69 (7.5)		16 (5.1)	61 (8.3)	
Household net ownership						
No nets in household	79 (41.2)	200 (21.9)	< 0.001	103 (32.5)	164 (22.2)	< 0.001
≥ 1 ITN in household	113 (58.9)	715 (78.1)		214 (67.5)	574 (77.8)	
Net use on the previous night*						
Did not sleep under a net	89 (55.6)	321 (40.3)	< 0.001	128 (47.6)	267 (41.5)	0.092
Slept under a net	97 (44.4)	475 (59.7)		141 (52.4)	376 (58.5)	
Ownership and use of mosquito nets†						
Household does not have nets	61 (38.1)	160 (20.1)	< 0.001	78 (29.0)	132 (20.5)	0.021
Did not use nets the previous night, but ≥ 1 net in household	28 (17.5)	162 (20.4)		50 (18.6)	136 (21.2)	
Used nets on the previous night	71 (44.4)	474 (59.6)		141 (52.4)	375 (58.3)	

ITN = insecticide-treated net; RDT = rapid diagnostic test. A total of 1,107 participants had RDT data (192 RDT positive) and 1,055 had data describing the presence of recent exposure markers (319 positive).

* Data on net use on the previous night missing from 32 cases and 119 controls by RDT, and missing from 48 cases and 95 controls by recent exposure marker.

† Excludes individuals missing net use data (32 cases and 119 controls by RDT, and 48 cases and 95 controls by recent exposure marker).

A total of 244 individuals (22%) reported any overnight travel outside their *section communale* (subdivision of administrative commune) in the previous 12 months, with 126 traveling in the 2 months before enrolment in the study. Further details of frequent destinations, reasons for travel, and mode of travel are described in Supplemental Table 3 and Supplemental Figures 2 and 3. Travelers included males and females of all age-groups, traveling to destinations including Port-au-Prince (43% of trips; incidence 0.3 per 1,000 in 2017), Les Irois (17% of trips; incidence 65.2 per 1,000 in 2017), and Les Cayes (15% of trips; incidence 1.7 per 1,000 in 2017). All reported travel was to destinations with very low transmission (< 100 per 1000 annual parasite incidence (API), WHO classification^25^), whereas 73% of trips were to destinations with annual incidence < 10 per 1,000. The reported use of mosquito nets during travel was low (travelers reported sleeping under an ITN on 28% of trips).

### Spatial clustering of cases.

**Figure 3.**
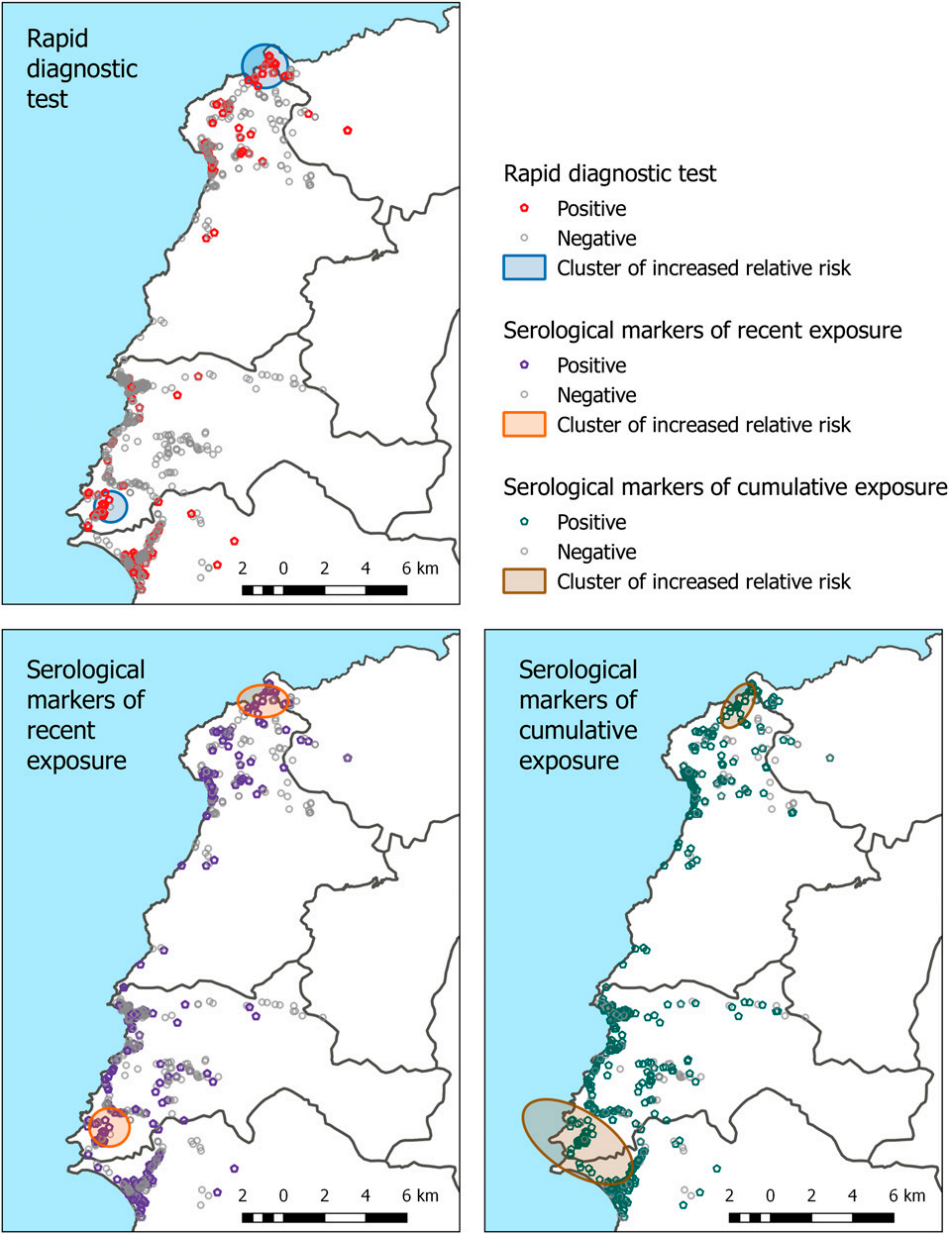
Maps of statistically significant (*P* < 0.05) clusters of rapid diagnostic test (RDT) positivity (upper left), recent exposure to *Plasmodium falciparum* (lower left), and cumulative exposure to *P. falciparum* (lower right) identified from the Bernoulli model using SaTScan.

Household GPS coordinates were recorded for all but one participant. Statistically significant clusters of RDT positives (Figure 3) were found in the north of the study area in La Seringue (risk ratio [RR] 3.49, *P* < 0.001) and in the southwest in Labite (RR = 4.23, *P* < 0.001). Clusters were identified in similar areas for markers of recent exposure (RR = 2.21, *P* = 0.036 and RR = 2.66, *P* < 0.001) and for markers of cumulative exposure (RR = 1.53, *P* = 0.002 and RR = 1.39, *P* = 0.014). The consistency in these clusters for outcomes relating to different periods of malaria exposure (several weeks for RDT, several months for serological markers of recent exposure, and several years for serological markers of cumulative exposure) suggests that the pattern of transmission has remained relatively stable over time.

### Risk and protective factors for malaria.

In univariate analysis, individuals in households with at least one ITN had lower odds of RDT positivity (OR: 0.46, *P* < 0.001), similarly being in a household where some or all individuals slept under a net had significantly lower odds of RDT positivity than households where no one used a net (OR: 0.58, *P* = 0.006 and OR: 0.42, *P* < 0.001, respectively). There was evidence of increased risk of RDT positivity among those living in households with no walls or walls of palm or bamboo compared with houses with cement block or stone walls (OR: 1.84, *P* = 0.006). In univariate analysis, individuals who were further from public health facilities, further from cities, at elevations below 100 m, and in forested areas all had higher odds of RDT positivity. Univariate risk factors of serological markers of recent exposure were similar to factors crudely associated with RDT positivity, including household ITN ownership, ITN use, and household construction. Those further from health facilities, further from cities, and in forested areas were also at increased risk of having serological markers of recent exposure. Travel was not a risk factor for RDT positivity or serological markers of recent exposure in univariate analysis.

In the final multivariate model, strong associations were found between RDT positivity and walking time to the nearest health facility, if the household was in a forested area, a binary variable identifying households with natural material or no walls (against all other types), and ownership and use of mosquito nets (Table 2). Associations were also seen between RDT positivity and the use of mosquito coil in the previous week, and if the respondent spent time outside their household after sunset. Gender, age-group, recruiting health facility, predicted malaria incidence at household location, and transmission season were preselected for inclusion in the RDT model.

**Table 2:** Adjusted odds ratios describing risk factors for RDT positivity in treatment-seeking febrile population (*N* = 941)

	Adjusted OR	95% CI	*P*-value
Age-group of participant (years)			
< 5	1.00	–	–
5–14	2.34	1.29, 4.26	0.005
15–29	2.18	1.17, 4.06	0.015
30–45	1.37	0.68, 2.76	0.379
> 45	2.13	0.92, 4.91	0.077
Female participant	0.88	0.60, 1.29	0.512
Recruiting facility			
Les Irois	1.00	–	–
Mandou	0.40	0.19, 0.81	0.011
Anse d’Hainault	0.21	0.10, 0.44	< 0.001
Petite Rivière	0.31	0.13, 0.72	0.006
Predicted malaria incidence per 1,000 population per year at household location*	1.00	0.99, 1.01	0.538
Transmission season			
Off-peak	1.00	–	–
Peak	2.65	1.69, 4.15	< 0.001
Ownership and use of mosquito nets			
Household does not have nets	1.00	–	–
Did not use nets the previous night, but ≥ 1 net in household	0.52	0.30, 0.89	0.018
Used nets on the previous night	0.57	0.37, 0.90	0.015
Household used mosquito coil in previous week	0.63	0.40, 1.00	0.049
Stayed outside the household after sunset	1.53	0.98, 2.38	0.060
Household has no walls or palm leaf walls	1.90	1.21, 3.00	0.005
Walking time to the nearest public health facility†	1.54	1.11, 2.12	0.009
Forest cover at household location	2.24	1.32, 3.80	0.003

RDT = rapid diagnostic test. Age-group, gender, and recruiting facility were preselected for inclusion in the model, and predicted malaria incidence variable was used to adjust for differences in underlying transmission intensity.

* Predicted model generated by the Malaria Atlas Project (MAP) to estimate the median incidence of malaria per 1,000 population per year.

† Where one unit relates to a 10-minute increase in walking time.

Household construction and remoteness from a health facility were found to be important risk factors after adjusting for confounders; participants from households with no walls or only palm or bamboo walls had higher odds of RDT positivity than those in all other household types (adjusted odds ratio [AOR]: 1.90, 95% CI: 1.21–3.00, *P* = 0.005), whereas odds of RDT positivity were also found to increase with increased remoteness from a health facility (AOR: 1.54 for a 10 minutes increase in time to walk to the facility, 95% CI: 1.11–2.12, *P* = 0.009).

The RDT model found a protective effect of both use of nets and living in a household with nets even if the individual did not use a net on the previous night (Table 2). After accounting for confounding factors, including underlying geographic risk, individuals using a net on the previous night had lower odds of RDT positivity than individuals in households with no nets (AOR: 0.57, 95% CI: 0.37–0.90, *P* = 0.015), as did individuals who did not use a net but live in a household with at least one ITN (AOR: 0.52, 95% CI: 0.30–0.89, *P* = 0.018). There was weak evidence for increased odds of RDT positivity among those spending time outside the household after sunset (AOR: 1.53, 95% CI: 0.98–2.38, *P* = 0.060) and weak evidence suggesting a protective effect of the use of mosquito coils (AOR: 0.63, 95% CI: 0.40–1.00, *P* = 0.049). Full output from models stratified by peak or nonpeak transmission season is presented in Supplemental Table 4. Briefly, residing close to a health facility and all household members using nets were protective in both seasons. In the peak season, household construction and spending time outside after dusk were also retained in models, whereas in the off-peak season, the use of mosquito coils and livestock ownership were protective, and there was weak evidence that recent travel was associated with RDT positivity.

The multivariate model with markers of recent exposure to *P. falciparum* as the primary outcome found that walking time to the nearest health facility and if the household was in a forested area were important risk factors (Table 3). A protective association between the reported use of mosquito coil in the previous week and markers of recent exposure was also identified. The final model preselected gender, age-group, recruiting health facility, and predicted malaria incidence at household location for inclusion. In contrast to the RDT model, there was no evidence for any association between any net ownership or use variables with odds of seropositivity to markers of recent *P. falciparum* exposure, after accounting for confounders. Similarly, there was no association with household construction or spending time outside the household. The only factors found to be associated with seropositivity were the use of mosquito coils (AOR: 0.69, 95% CI: 0.59–0.98, *P* = 0.037), time to walk to the nearest facility (AOR: 1.41 for 10 minutes increase, 95% CI: 1.08–1.83, *P* = 0.010), and forest cover (AOR: 1.93, 95% CI: 1.24–2.98, *P* = 0.003), all of which showed similar strength and direction of association as in the RDT positivity model.

**Table 3:** Adjusted odds ratios describing risk factors for marker of recent exposure to *Plasmodium* (positivity to antigen target Etramp 5 Ag 1 in multiplex bead-based assay) in the treatment-seeking febrile population (*N* = 916)

	Adjusted OR	95% CI	*P*-value
Age-group of participant (years)			
< 5	1.00	–	–
5–14	2.33	1.43, 3.80	0.001
15–29	2.53	1.55, 4.14	< 0.001
30–45	2.95	1.75, 4.96	< 0.001
> 45	3.99	2.16, 7.39	< 0.001
Female participant	1.09	0.81, 1.48	0.568
Recruiting facility			
Les Irois	1.00	–	–
Mandou	0.88	0.50, 1.56	0.669
Anse d’Hainault	0.51	0.31, 0.84	0.009
Petite Rivière	0.55	0.28, 1.09	0.086
Predicted malaria incidence per 1,000 population per year at household location*	1.00	0.99, 1.01	0.804
Household used mosquito coil in previous week	0.69	0.49, 0.98	0.037
Walking time to nearest public health facility†	1.41	1.08, 1.83	0.010
Forest cover at household location	1.93	1.24, 2.98	0.003

Age-group, gender, and recruiting facility were preselected for inclusion in the model.

* Predicted model generated by the Malaria Atlas Project to estimate the median incidence of malaria per 1,000 population per year.

† Where one unit relates to a 10-minute increase in walking time.

## DISCUSSION

This case–control study aimed to identify key demographic, behavioral, and geographic risk factors for malaria among febrile, treatment-seeking individuals in the Grand’Anse department of Haiti, an area targeting elimination by 2025. Current infection was assessed by RDT, and MBA was used to detect serological markers of recent and cumulative exposure to *P. falciparum*. This study indicates that, in this area, an individuals’ risk of malaria is primarily driven by their location, not by specific occupational or demographic characteristics. Cluster analysis indicates that geographic foci of increased risk are stable over time. Access to treatment is an important predictor, with those living far from health facilities found to have increased risk of malaria. Furthermore, this study provides evidence of a protective association between ownership and the use of ITNs and reduced odds of RDT positivity, after accounting for underlying transmission intensity; however, no association was found between ITN ownership and use and markers of recent exposure to *P. falciparum*. The results also indicate that those in households with walls made of unsealed natural materials or without any walls are at increased risk, suggesting some indoor biting by local vectors.

Spatial analysis identified clusters of increased RR of malaria that were broadly consistent for each of the main outcome variables. Clusters were between 2 km^2^ and 10 km^2^ in size, of a relevant scale for intervention targeting to specific hamlets, villages, or towns. The consistency of these clusters identified by current RDT infection and by markers of recent and cumulative exposure to *P. falciparum* indicates that these are stable locations of increased malaria transmission, relative to the surrounding area. The two main areas of increased RR of malaria were the Le Seringue area to the north of the study area and the village of Labite in the southern part of the study area. These villages are less accessible than most of the rest of the study area, requiring transport by motorcycle or by foot, and do not have a permanent public health clinic, instead being served intermittently by outreach teams from the closest health facilities (Petite Rivière and Mandou). Multivariate models found that time to walk to the nearest heath facility was associated with both RDT positivity and the presence of serological markers of recent exposure to *P. falciparum*, suggesting that malaria transmission persists in these remote communities. Transmission persistence in these communities is hypothesized to be a consequence of both lower health-seeking behavior and environmental suitability. A similar finding of increased risk of malaria in remote areas was identified in easy access group surveys in Artibonite Department, central Haiti.^11^

A number of different factors were associated with odds of malaria in the coastal Grand’Anse area, which will be important for informing malaria elimination programming. First, not sleeping under a net but living in a household with at least one ITN, and use of an ITN on the previous night were associated with reduced odds of RDT positivity. Net ownership and use were not associated with serologic markers of recent exposure to *P. falciparum*. A previous case–control study in Haiti found that consistent use of ITNs (defined as ITN use on all 14 nights in the 2 weeks before the onset of illness) was not associated with a protective effect against malaria, but in the context of very low levels of consistent net use.^9^ The low ITN use (13% consistent use) in the 2012–2014 study was likely insufficient for a “community effect” and suppression of the vector population. The authors also found no personal protective effect of ITN use,^9^ a not uncommon finding in case–control studies, even in areas where randomized controlled trials have demonstrated impact.^26,27^ An ITN distribution took place in Grand’Anse in June 2017 (approximately 1 year before the current study), and 77% of individuals in the current study reported that their household owned at least one net, with 95.7% of all nets confirmed to be long-lasting insecticidal nets from WHO-prequalified brands. However, when considering access to ITNs among all household members,^28^ only 54% of the study population had access to an ITN, indicating that insufficient nets are available to protect all of the population at risk of malaria. It is likely that in the current study net coverage was insufficient for a wider community effect, but household-level and individual-level associations between ITNs and reduced odds of RDT positivity were observed. We hypothesize that ITNs provided a repellent effect against mosquitoes in the household; however, the evidence of ITN repellency is mixed,^29^ with potential that ITN repellent effects could divert mosquitoes toward unprotected individuals when not all household members are protected by a net.^30^ Furthermore, as all communes in Grand’Anse were targeted in the 2017 net distribution, there is little expected endogeneity from ITNs being more available in areas with higher malaria transmission risk. Although the recent exposure marker has been validated in children,^31^ there are limited data on how long this marker persists in adults. Consequently, the recent exposure marker could include exposure to *P. falciparum* more than one year ago in adults. This may include the period before the 2017 mass ITN distribution, potentially contributing to the lack of association between ITNs and recent exposure marker.

An additional difference between the two case–control studies was in the choice of indicator to summarize ITN use by individuals. Whereas the 2012–2014 study used a binary classification of use on all previous 14 nights before illness onset, as well as the common indicator of bednet use the previous night, the current study used only reported net use on the previous night. Net use on the previous night is the standard ITN use indicator recommended by Roll Back Malaria, but does assume that net use on the previous night is representative of use during the exposure period of interest.^32^ As such, it is possible that ITN use was overestimated in the current study. Seasonal changes in ITN use may also partly explain the lack of association between recent exposure markers and ITN ownership or use because reported ITN use on the previous night may not be representative of the time period captured by the recent exposure marker.

Household construction, specifically wall material, was found to be relevant in the RDT model. Individuals living in household which had either no wall structures or walls that were made of palm leaf or bamboo were found to have increased odds of RDT positivity. This type of open house structure, or the use of materials such as palm leaf or bamboo which leave large gaps in the wall, permits easy entry of mosquitoes to the household. *Anopheles albimanus*, the primary malaria vector in Haiti, is understood to be generally exophilic,^3^ but this type of open or natural material wall structure adds complexity to understanding endophilic versus exophilic vector behavior. The presence of households without walls or with walls made of unsealed natural or improvised materials may be partly attributable to the Grand’Anse region being severely affected by Hurricane Matthew in October 2016. A study investigating associations between household construction and malaria risk in sub-Saharan Africa found that modern housing (built with finished wall, roof, and floor materials) was associated with a 9–14% reduction in odds of malaria compared with traditional housing (all other materials),^33^ and there is interest in mapping changes in housing structure over time to investigate the potential impact of improved housing on malaria transmission.^34^ Modeling suggests that the protective effect of ITNs against *An. albimanus* in Haiti would be lower than ITN effect against African vectors but that ITN use in Haiti could prevent approximately half of *An. albimanus* bites, whereas house screening could be a very effective intervention in Grand’Anse if people are inside during peak biting periods.^35^ Entomological data collected in the current case–control study describing indoor and outdoor vector density and blood meal analysis will be detailed in a separate publication (Joseph et al., in preparation).

This study did not find evidence for any specific occupational risk factors. The study design did not allow investigation of age as a risk factor, but a lower proportion of children younger than 5 years was observed to be RDT positive or have markers of recent exposure than other age-groups. As malaria transmission reduces, the causes of acute febrile illness become increasingly varied, particularly among children.^36,37^ There was some evidence of behavioral risk factors; individuals who reported that they stayed outside their households after sunset were found to have increased odds of RDT positivity (but no association with markers of recent exposure). Although the interview asked what time individuals usually enter their household at night, no further information was collected on their location or activities outside the household in the evening. A recent review found substantial variation in types and locations of human nighttime activity that may be relevant to malaria exposure across different settings in sub-Saharan Africa.^38^

Although data were collected describing travel outside of the *section communale* for at least one night, travel was not found to be associated with RDT positivity or markers of recent exposure. The lack of association between travel and infection or exposure remained even if excluding travel to the capital, Port-au-Prince, understood to have very low malaria risk. In contrast to other settings targeting elimination,^39,40^ the Grand’Anse area has potential to act as a “source” rather than “sink” of cases because most infections appear to be locally acquired and travel is not a risk factor for malaria. Preliminary molecular evidence supports the conclusion that malaria is locally acquired; parasite populations in each region of Haiti have distinct genetic profiles, with little evidence of parasite movement between regions (S. Volkman, personal communication).

The study had a number of limitations. First, the target sample size was not reached as a result of a nationwide drop in malaria cases during 2018. Although this smaller sample size will have reduced the power of the study, it was possible to identify significant risk factors. Furthermore, our sample size for the multivariate models was slightly reduced as a result of missing information about the case or control and their reported ITN use collected during the follow-up interview. Multivariate models included predicted annual malaria incidence at participants’ household location as a covariate, estimates generated by a geostatistical model developed by the Malaria Atlas Project. Although including predicted incidence provides a simple approach to account for underlying transmission intensity, there is a risk of multicollinearity if the same covariates are included in the modeled incidence surface and in our models exploring risk factors.

The second main limitation is that our study design was limited to individuals seeking treatment from four public health facilities, and it is known that treatment seeking for diagnosis and treatment of fever is low.^2,41^ This may have biased our results if the level of potential risk factors differs between the treatment-seeking and nontreatment-seeking populations. Cross-sectional surveys in Haiti will complement these findings and may identify any additional risk factors which are not prevalent among the treatment-seeking population (Hamre et al., in preparation).^11^ The recruitment strategy whereby clinicians identified individuals eligible to participate in the study (febrile individuals tested by RDT) was used to minimize disruption at the facility compared with the study team conducting systematic screening of all patients; however, there is potential for bias if clinicians did not refer all eligible individuals to the study team. Furthermore, our purposive selection of study facilities excluded the most remote health facilities, which may have underestimated the role of distance to facility as a predictor of malaria risk. Finally, the method used to identify clusters of increased malaria risk was limited to circular or ellipsoid shapes. It is known that clusters of increased risk may take other shapes, for example, following rivers, coastlines, or other geographical features, and therefore spatially explicit modeling could provide more detail in identification of areas of increased malaria risk.

This study has demonstrated that after accounting for underlying malaria transmission (from a predictive model of malaria incidence), housing structure and access to ITNs are important factors that are associated with risk of malaria in Grand’Anse when assessed by RDT. There is additional evidence that suggests time spent outside the household in the evening and increasing distance from health facilities may be associated with malaria; however, additional data may be required to confirm these findings. There remain unanswered questions about the relative importance of indoor versus outdoor exposure to vector biting, which may support prioritization of intervention strategies. Ongoing entomological studies in the Grand’Anse are expected to shed some light on the relative abundance of malaria vectors inside and outside, the proportions of human and animal blood meals among relevant *Anopheles* species, and the sporozoite rate.

## CONCLUSION

Coastal areas of Grand’Anse targeting malaria elimination appear to have temporally stable foci of increased risk compared with the wider study area, and no major demographic, behavioral, or occupational risk factors were identified in this study. Malaria risk in Grand’Anse is largely a result of the setting where an individual lives; remoteness from health facilities, housing with walls that permit easy mosquito entry, and being in a forested area were all associated with increased odds of RDT positivity. This study also provides some evidence of a protective effect of ITNs, indicating that ITNs may be an appropriate vector control intervention in these coastal areas of Grand’Anse.

## Supplemental tables and figures

Supplemental materials
