# Supplementary material for: Risk Factors for Malaria Infection and Seropositivity in the Elimination Area of Grand’Anse, Haiti: A Case–Control Study among Febrile Individuals Seeking Treatment at Public Health Facilities
Source: Am J Trop Med Hyg. 2020 May 26;103(2):767–77. doi: 10.4269/ajtmh.20-0097 (PMC7410432; doi:10.4269/ajtmh.20-0097)

**Supplementary material**

**Ashton et al. “Risk factors for malaria infection and seropositivity in the elimination area of Grand’Anse, Haiti: A case control study among febrile individuals seeking treatment at public health facilities”**

**Supplementary figure 1.** Flowchart describing participant enrolment in the case control study


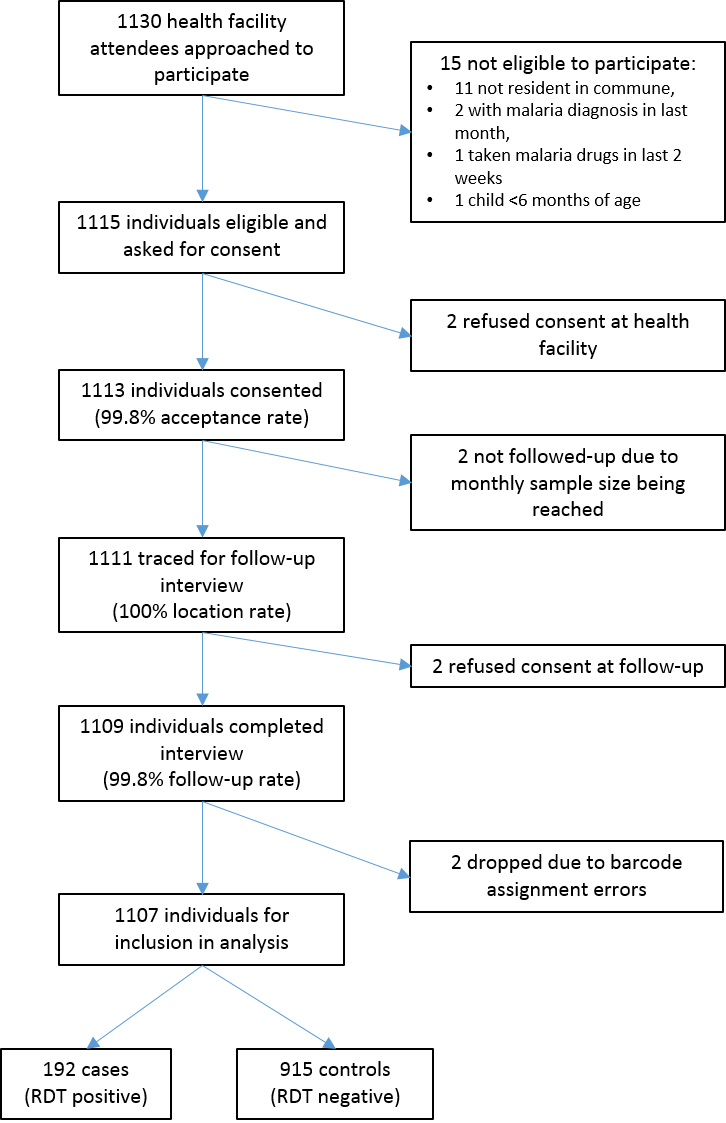


**Supplementary Table 1** Demographic characteristics of study participants and Chi-squared p-values using alternative case-control classification: presence of antibodies representing cumulative (historical) exposure to *P. falciparum* (positivity to either PfAMA1 or PfMSP1-19 in multiplex bead-based assay (N=1055).

|  | **Cumulative exposure markers** | | |
| --- | --- | --- | --- |
|  | **Case** | **Control** |  |
|  | **n (%)** | **n (%)** | **p** |
| Sex of case/control |  |  |  |
| Male | 318 (45.4) | 169 (47.7) | 0.465 |
| Female | 383 (54.6) | 185 (52.3) |  |
| Age group |  |  |  |
| <5 | 60 (8.6) | 171 (48.3) | <0.001 |
| 5-14 | 176 (25.1) | 107 (30.2) |  |
| 15-29 | 222 (31.7) | 49 (13.8) |  |
| 30-44 | 161 (23.0) | 18 (5.1) |  |
| 45+ | 82 (11.7) | 9 (2.5) |  |
| Recruiting facility |  |  |  |
| Les Irois | 148 (21.1) | 75 (21.2) | 0.007 |
| Mandou | 148 (21.1) | 58 (16.4) |  |
| Jean Baptiste de l'Anse d'Hainault | 175 (25.0) | 233 (34.5) |  |
| Petite Rivière | 230 (32.8) | 99 (38.0) |  |
| Time lived in the current community |  |  |  |
| <1 year | 9 (1.3) | 8 (2.3) | <0.001 |
| 1-2 years | 95 (13.6) | 74 (20.9) |  |
| 3-4 years | 38 (5.4) | 31 (8.8) |  |
| 5-9 years | 48 (6.9) | 18 (5.1) |  |
| 10+ years | 95 (13.6) | 13 (3.7) |  |
| Whole life (all ages) | 416 (59.3) | 210 (59.3) |  |
| Any travel in previous 12 months |  |  |  |
| Yes | 160 (22.8) | 77 (21.8) | 0.693 |
| No | 541 (77.2) | 277 (78.3) |  |
| Livestock ownership |  |  |  |
| Yes | 463 (66.1) | 210 (59.3) | 0.032 |
| No | 238 (34.0) | 144 (40.7) |  |
| Roof material |  |  |  |
| Thatch/palm leaf/bamboo | 20 (2.9) | 11 (3.1) | 0.197 |
| Canvas/tent | 176 (25.1) | 69 (19.5) |  |
| Iron sheets | 447 (63.8) | 236 (66.7) |  |
| Tiles/cement | 54 (7.7) | 37 (10.5) |  |
| Other | 4 (0.6) | 1 (0.3) |  |
| Wall material |  |  |  |
| No walls or palm leaf/bamboo | 136 (19.4) | 48 (13.6) | 0.043 |
| Bamboo & mud or stone & mud | 197 (28.1) | 106 (29.9) |  |
| Wood plank or salvaged wood | 28 (4.0) | 27 (7.6) |  |
| Canvas or tent | 41 (5.9) | 25 (7.1) |  |
| Metal sheet | 9 (1.3) | 5 (1.4) |  |
| Cement block or stone & cement | 290 (41.4) | 143 (40.4) |  |
| Occupation of case/control |  |  |  |
| <16 years | 256 (36.5) | 283 (79.9) | <0.001 |
| Student | 102 (14.6) | 29 (8.2) |  |
| Agriculture or fishing | 123 (17.6) | 6 (1.7) |  |
| Day labor | 24 (3.4) | 7 (2.0) |  |
| Shop keeper | 133 (19.0) | 15 (4.2) |  |
| Other | 63 (9.0) | 14 (4.0) |  |
| Household net ownership |  |  |  |
| No nets in household | 193 (27.5) | 74 (20.9) | 0.019 |
| ≥1 ITN in household | 508 (72.5) | 280 (79.1) |  |
| Net use on previous night^1^ |  |  |  |
| Did not sleep under net | 282 (46.2) | 113 (37.4) | 0.011 |
| Slept under net | 328 (53.8) | 189 (62.6) |  |
| Ownership and use of mosquito nets^2^ |  |  |  |
| Household doesn’t have nets | 152 (24.9) | 58 (19.2) | 0.033 |
| Didn’t use net previous night, but ≥1 net in household | 131 (21.5) | 55 (18.2) |  |
| Used net on previous night | 327 (53.6) | 189 (62.6) |  |

^1^Data on net use in previous night missing from 91 cases and 52 controls by cumulative exposure marker

^2^Excludes individuals missing net use data: 91 cases and 52 controls by cumulative exposure marker

ITN: insecticide-treated net

**Supplementary table 2:** Univariate associations between case-control status (by rapid diagnostic test (RDT) and by markers of recent exposure to *P. falciparum*) and key demographic and household characteristics. The denominator for RDT outcome is 1107 individuals (except insecticide-treated net (ITN) use variables, where N=961). The denominator for recent exposure markers outcome is 1070 (except ITN user variables where N=923).

|  | **Rapid diagnostic test** | | | **Recent exposure markers** | |
| --- | --- | --- | --- | --- | --- |
|  | **Crude OR**  **(95% CI)** | **p-value** | **Crude OR**  **(95% CI)** | | **p-value** |
| *Individual-level variables* |  |  |  | |  |
| Occupation of case/control |  |  |  | |  |
| <16 years | 1.25 (0.41,3.86) | 0.697 | 0.72 (0.26,2.00) | | 0.527 |
| Student | 1.37 (0.66,2.81) | 0.397 | 1.07 (0.57,2.00) | | 0.834 |
| Agriculture or fishing | 1.00 | - | 1.00 | | - |
| Day labor | 1.80 (0.69,4.69) | 0.232 | 0.71 (0.29,1.70) | | 0.437 |
| Shop keeper | 0.94 (0.48,1.87) | 0.869 | 1.20 (0.70,2.04) | | 0.504 |
| Other | 0.98 (0.41,2.33) | 0.957 | 0.58 (0.29,1.15) | | 0.116 |
| Overnight travel in last 2 months | 1.13 (0.68,1.90) | 0.633 | 0.96 (0.62,1.50) | | 0.868 |
| Overnight travel in the last 2 months, excluding travel to the Port-au-Prince area^1^ | 1.38 (0.77, 2.46) | 0.275 | 1.05 (0.62, 1.78) | | 0.856 |
| Enter household after 7pm | 1.15 (0.79,1.66) | 0.471 | 0.95 (0.70,1.30) | | 0.760 |
| Exit household before dawn | 1.28 (0.61,2.67) | 0.509 | 1.02 (0.53,1.95) | | 0.956 |
| Slept under net last night | 0.68 (0.48,0.98) | **0.037** | 0.91 (0.67,1.23) | | 0.549 |
| *Household level variables* |  |  |  | |  |
| Household has ≥1 mosquito net | 0.46 (0.33,0.65) | **<0.001** | 0.66 (0.48,0.89) | | **0.007** |
| Household has ≥1 net for 2 people | 0.67 (0.46,0.98) | **0.040** | 0.77 (0.57,1.04) | | 0.093 |
| How many people in case/control’s household slept under a net last night |  |  |  | |  |
| None | 1.00 | **-** | 1.00 | | - |
| Some | 0.58 (0.39,0.86) | **0.006** | 0.83 (0.59,1.16) | | 0.279 |
| All | 0.42 (0.27,0.64) | **<0.001** | 0.69 (0.49,0.98) | | **0.038** |
| Proportion of people in case/control’s household who slept under a net last night | 0.47 (0.31,0.70) | **<0.001** | 0.70 (0.51,0.98) | | **0.036** |
| Number people in case/control’s household who did not sleep under net previous night | 1.12 (1.05,1.19) | **<0.001** | 1.08 (1.02,1.14) | | **0.007** |
| Household uses mosquito coil or insecticide spray | 0.59 (0.40,0.88) | **0.009** | 0.65 (0.47,0.90) | | **0.010** |
| Household uses domestic insecticide spray | 1.70 (0.70,4.12) | 0.243 | 1.09 (0.50,2.43) | | 0.832 |
| Female head of household | 0.78 (0.55,1.11) | 0.175 | 1.03 (0.77,1.38) | | 0.848 |
| Head of household never attended school | 1.21 (0.86,1.70) | 0.284 | 1.10 (0.82,1.47) | | 0.533 |
| Roof material |  |  |  | |  |
| Thatch/palm leaf/bamboo | 1.43 (0.59,3.50) | 0.430 | 1.30 (0.59,2.85) | | 0.509 |
| Canvas/tent | 1.45 (1.01,2.10) | **0.045** | 1.14 (0.82,1.57) | | 0.433 |
| Iron sheets | 1.00 | - | 1.00 | | - |
| Tiles/cement/reinforced concrete | 0.55 (0.26,1.16) | 0.119 | 0.58 (0.33,1.03) | | 0.065 |
| Other | 0.88 (0.09,8.31) | 0.911 | 0.44 (0.05,4.07) | | 0.467 |
| Household wall material |  |  |  | |  |
| No walls or palm leaf/bamboo | 1.84 (1.19,2.86) | **0.006** | 1.65 (1.11,2.46) | | **0.013** |
| Bamboo & mud or stone & mud | 1.10 (0.73,1.66) | 0.658 | 1.33 (0.94,1.88) | | 0.104 |
| Wood plank/salvaged wood | 2.18 (0.85,5.56) | 0.103 | 1.27 (0.61,2.66) | | 0.529 |
| Canvas/tent | 0.73 (0.27,1.94) | 0.523 | 1.39 (0.74,2.60) | | 0.302 |
| Metal sheet | 1.46 (0.28,7.70) | 0.659 | 1.90 (0.55,6.61) | | 0.313 |
| Cement block or stone & cement | 1.00 | - | 1.00 | | - |
| Household has open/partially open eaves | 0.93 (0.54,1.60) | 0.799 | 1.31 (0.84,2.03) | | 0.231 |
| Household main building have a ceiling? |  |  |  | |  |
| No ceiling | 1.00 | - | 1.00 | | - |
| Partial/poorly sealed/worn out ceiling | 1.05 (0.51,2.16) | 0.900 | 1.06 (0.61,1.82) | | 0.846 |
| Complete and sealed ceiling | 0.35 (0.10,1.20) | 0.096 | 0.55 (0.22,1.41) | | 0.213 |
| Household windows and airbricks screened? |  |  |  | |  |
| No | 1.00 | - | 1.00 | | - |
| Partially screened | 1.17 (0.55,2.46) | 0.687 | 1.35 (0.72,2.51) | | 0.345 |
| Completely screened | 0.71 (0.09,5.77) | 0.751 | 0.27 (0.03,2.15) | | 0.218 |
| Household owns any livestock | 1.08 (0.72,1.60) | 0.718 | 1.18 (0.85,1.63) | | 0.335 |
| Livestock kept close to household | 1.01 (0.56,1.84) | 0.965 | 1.37 (0.85,2.20) | | 0.200 |
| Household wealth |  |  |  | |  |
| Poorest | 1.73 (1.00,2.97) | **0.049** | 1.76 (1.22,2.75) | | **0.014** |
| 2^nd^ | 1.36 (0.79,2.33) | 0.262 | 1.64 (1.06,2.54) | | **0.025** |
| Median | 1.47 (0.76,2.85) | 0.250 | 1.89 (0.95,3.00) | | 0.074 |
| 4^th^ | 1.34 (0.76,2.34) | 0.310 | 1.15 (0.72,1.84) | | 0.562 |
| Richest | 1.00 | - | 1.00 | | - |
| *Environmental / location data* |  |  |  | |  |
| Walking time to nearest public health facility^2^ | 1.58 (1.33,1.88) | **<0.001** | 1.42 (1.21,1.68) | | **<0.001** |
| Travel time (mins) to nearest city |  |  |  | |  |
| ≤2.5 hours | 1.00 | - | 1.00 | | **-** |
| >2.5 hours | 2.42 (1.12,5.27) | **0.025** | 1.64 (1.01,2.68) | | **0.045** |
| Probability of population living the area of seeking treatment at public health facility^3^ | 0.67 (0.57,0.79) | **<0.001** | 0.84 (0.72,0.96) | | **0.013** |
| Elevation |  |  |  | |  |
| <10m | 0.39 (0.20,0.79) | **0.008** | 0.71 (0.39,1.29) | | 0.261 |
| 10-100m | 0.45 (0.29,0.70) | **<0.001** | 0.76 (0.52,1.11) | | 0.156 |
| >100m | 1.00 | **-** | 1.00 | | - |
| Forest cover at household location (binary) | 1.89 (1.25,2.85) | **0.002** | 1.58 (1.09,2.29) | | 0.016 |
| Cropland at household location (binary) | 0.76 (0.50,1.15) | 0.191 | 0.94 (0.67,1.32) | | 0.721 |
| Rain in month prior to diagnosis |  |  | - | | - |
| ≤80 mm | 1.00 | - | - | | - |
| >80 mm | 2.69 (1.82,3.99) | **<0.001** | - | | - |
| Rain 2 months prior to diagnosis |  |  | - | | - |
| ≤80 mm | 1.00 | - | - | | - |
| >80 mm | 0.95 (0.68,1.35) | 0.795 | - | | - |
| NDVI same month^3^ | 1.46 (1.15,1.84) | **0.002** | - | | - |
| NDVI previous month |  |  | - | | - |
| <0.4 | 1.00 | - | - | | - |
| 0.4-0.78 | 1.06 (0.59,1.92) | 0.842 | - | | - |
| >0.78 | 1.01 (0.46,2.20) | 0.987 | - | | - |
| NDVI 2 months before diagnosis^3^ | 1.39 (1.12,1.74) | **0.003** | - | | - |
| Predicted malaria incidence per 1000 population per year at household location^4^ |  |  |  | |  |
| <25 cases/1000 population | 1.00 | - | 1.00 | | - |
| 20-99 cases/1000 population | 1.37 (0.77,2.46) | 0.287 | 1.63 (1.09,2.45) | | **0.018** |
| ≥100 cases /1000 population | 2.42 (1.11,5.29) | **0.026** | 2.29 (1.27,4.15) | | **0.006** |

^1^Crois-des-Bouquets, Carrefour, Cité-Soleil, Delmas, Pétion-ville, Tabarre, and Port-au-Prince communes classified as the greater Port-au-Prince area

^2^One unit indicates ten minutes;

^3^Normalized difference vegetation index, standardized to mean 0 and standard deviation 1.

^4^Predicted model generated by the Malaria Atlas Project (MAP) to estimate median incidence of malaria per 1000 population per year.

**Supplementary table 3**

Characterizing reported travel among case control study participants. 244 of 1107 (22%) participants reported any travel in the previous 12 months, with 126 (12%) reporting travel in the previous two months before enrolment. All 328 reported overnight trips by the 244 travelers are reported in the table below.

|  | **n** | **%** |
| --- | --- | --- |
| Destination |  |  |
| Within commune of residence | 59 | 18.0 |
| Within department (Grand Anse) | 34 | 10.4 |
| Beyond Grand Anse | 235 | 71.7 |
| Specific common destinations |  |  |
| Les Irois (town within study area) | 57 | 17.4 |
| Jérémie (department capital) | 18 | 5.5 |
| Tiburon (town in neighboring department) | 11 | 3.4 |
| Les Cayes (neighboring department capital) | 52 | 15.9 |
| Port-au-Prince area^1^ (capital city) | 141 | 43.0 |
| Other locations | 49 | 14.9 |
| Malaria incidence at destination commune^2^ |  |  |
| <1 per 1000 | 151 | 46.5 |
| 1 – 9.9 per 1000 | 85 | 26.2 |
| 10 – 49.9 per 1000 | 30 | 9.2 |
| ≥50 per 1000 | 59 | 18.2 |
| Purpose of trip |  |  |
| Visiting friends | 108 | 32.9 |
| Holiday | 79 | 24.1 |
| Business / trade / work | 72 | 22.0 |
| Medical | 22 | 6.7 |
| Marriage / funeral | 12 | 3.7 |
| Education | 9 | 2.7 |
| Farming | 8 | 2.4 |
| Other | 18 | 5.7 |
| Duration of travel |  |  |
| 1-3 nights | 89 | 27.1 |
| 4-7 nights | 64 | 19.5 |
| 8-14 nights | 45 | 13.7 |
| 15-29 nights | 45 | 13.7 |
| ≥ 30 nights | 85 | 25.9 |
| Method of travel |  |  |
| Bus | 193 | 58.8 |
| Motorcycle | 87 | 26.5 |
| Walking | 52 | 15.9 |
| Other | 16 | 4.9 |
| Any accompanying household members? |  |  |
| None | 193 | 58.8 |
| One | 87 | 26.5 |
| More than one | 48 | 14.6 |
| Slept under bednet while traveling? |  |  |
| Yes | 92 | 28.1 |
| Sometimes | 17 | 5.2 |
| No | 211 | 64.3 |
| Don’t know | 8 | 2.4 |

^1^Crois-des-Bouquets, Carrefour, Cité-Soleil, Delmas, Pétion-ville, Tabarre, and Port-au-Prince communes

^2^Incidence from routine surveillance data from 2017, aggregated to commune level

**Supplementary table 4**

Multivariate model output: risk factors for RDT positivity in off-peak season (prior to 4^th^ June, N=340), and in peak transmission season (from 4^th^ June onwards, N=611) among the treatment-seeking febrile population. Age group, sex and recruiting facility were included in models *a priori*.

|  | **Off-peak season (N=340)** | | | | **Peak season (N=611)** | | |
| --- | --- | --- | --- | --- | --- | --- | --- |
|  | **AOR** | **95% CI** | **p-value** | **AOR** | | **95% CI** | **p-value** |
| Age group of participant |  |  |  |  | |  |  |
| <5 years | 1.00 | - | - | 1.00 | | - | - |
| 5-14 years | 1.08 | 0.23, 5.12 | 0.919 | 2.89 | | 1.42, 5.88 | 0.003 |
| 15-29 years | 1.24 | 0.32, 4.84 | 0.752 | 2.64 | | 1.26, 5.54 | 0.010 |
| 30-45 years | 2.28 | 0.57, 9.21 | 0.247 | 1.18 | | 0.51, 2.75 | 0.699 |
| >45 years | 0.37 | 0.05, 2.63 | 0.321 | 2.78 | | 1.01, 7.64 | 0.048 |
| Female participant | 0.80 | 0.31, 2.04 | 0.639 | 0.90 | | 0.58, 1.39 | 0.637 |
| Recruiting facility |  |  |  |  | |  |  |
| ESPWA Les Irois | 1.00 | - | - | 1.00 | | - | - |
| SSPE Mandou | 1.65 | 0.37, 7.44 | 0.515 | 0.48 | | 0.22, 1.04 | 0.062 |
| Anse d’Hainault | 0.14 | 0.02, 1.15 | 0.067 | 0.30 | | 0.12, 0.78 | 0.013 |
| Petite Riviere | 0.83 | 0.23, 3.03 | 0.783 | 0.40 | | 0.23, 0.72 | 0.002 |
| Net use in household last night |  |  |  |  | |  |  |
| No-one used net | 1.00 | - | - | 1.00 | | - | - |
| Some used net | 0.75 | 0.27, 2.09 | 0.583 | 0.66 | | 0.39, 1.12 | 0.125 |
| All used net | 0.17 | 0.04, 0.69 | 0.013 | 0.56 | | 0.32, 0.98 | 0.041 |
| Household uses coil/spray | 0.09 | 0.02, 0.56 | 0.009 | - | | - | - |
| Household has any livestock | 0.30 | 0.10, 0.89 | 0.030 | - | | - | - |
| Any overnight travel in prior 2 months | 2.99 | 0.89, 10.03 | 0.076 | - | | - | - |
| Household has no walls or palm leaf walls | - | - | - | 1.69 | | 1.00, 2.86 | 0.052 |
| Stayed outside the household after sunset | - | - | - | 1.58 | | 0.96, 2.59 | 0.071 |
| Walking time in minutes to nearest public HF^2^ |  |  |  |  | |  |  |
| <6 minutes | 1.00 | - | - | 1.00 | | - | - |
| 6-15 minutes | 4.40 | 0.47, 41.48 | 0.196 | 2.48 | | 0.98, 6.24 | 0.054 |
| >15 minutes | 10.17 | 1.06, 97.98 | 0.045 | 2.74 | | 1.03, 7.25 | 0.043 |
| Cropland at household location | 0.12 | 0.03, 0.46 | 0.002 | - | | - | - |
| Rain in month prior to diagnosis (mm) |  |  |  |  | |  |  |
| < 80mm | 1.00 |  |  | - | | - | - |
| 80 - 120mm | 22.08 | 4.20, 116.2 | <0.001 | - | | - | - |
| >120 mm | 0.97 | 0.08, 11.50 | 0.979 | - | | - | - |
| NDVI 2 months before diagnosis | - | - | - | 34.13 | | 6.22, 187.2 | <0.001 |

^1^Urban area defined as population >50k, time in minutes

^2^Surface developed at 1km resolution, therefore should be interpreted as relative rather than absolute level of access to HFs

NDVI: normalized difference vegetation index


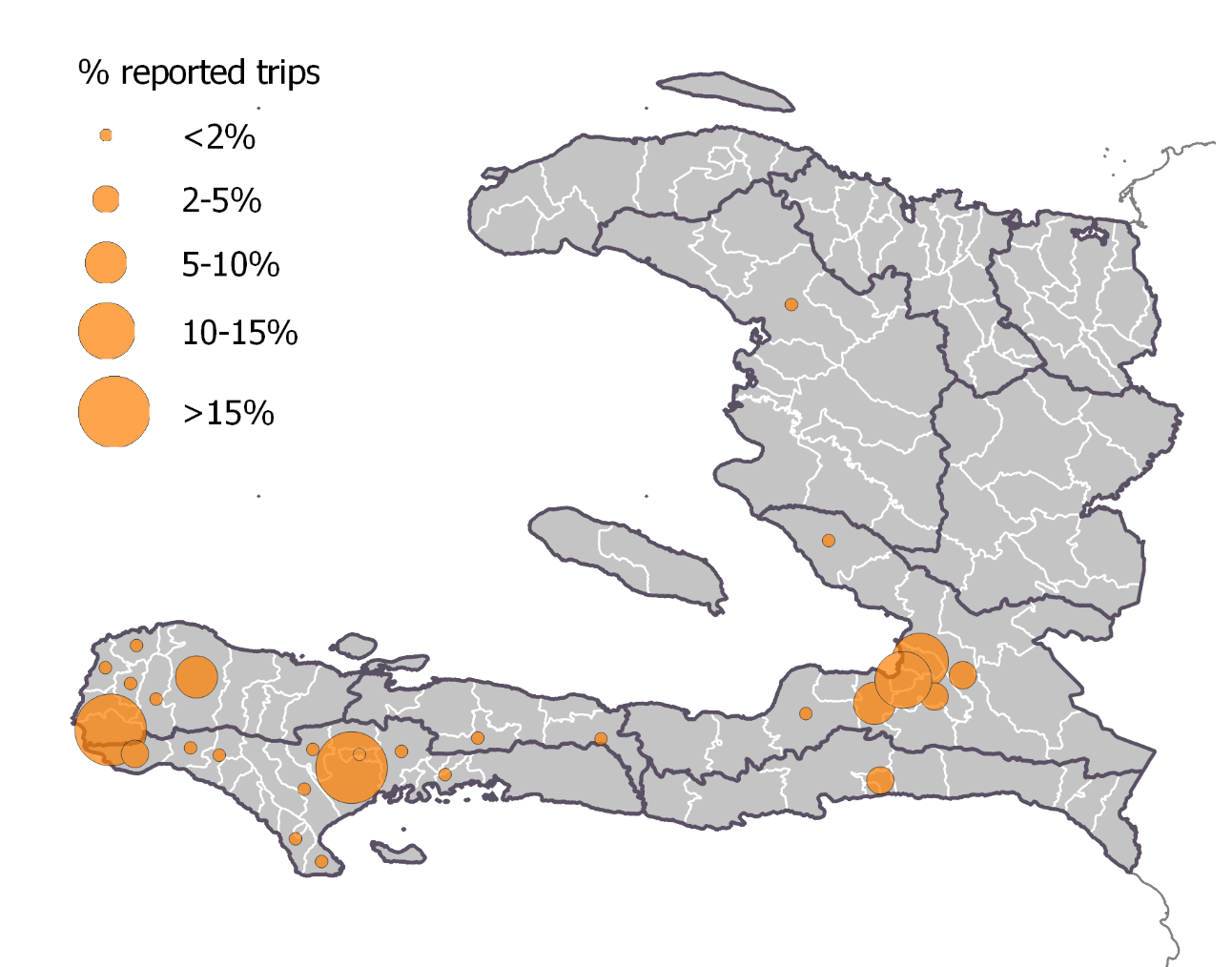
**Supplementary Figure 2**

Map presenting destinations (commune-level) of all overnight trips reported by participants in the

12 months, with size of marker indicating the proportion of all reported trips that had the specified commune as a destination.

**Supplementary Figures 3a, 3b, 3c, 3d**

Flow maps describing the destinations of all travel reported by participants in the 12 months that had a duration of at least one night, with inset map showing close-up view of local travel. Destination was coded to the commune-level, shown on the map by a centroid point for the commune. To simplify presentation, communes in the greater Port-au-Prince area (Carrefour, Cité-Soleil, Delmas, Pétion-ville, Port-au-Prince, and Tabarre) have been combined. Flow maps are presented separately for participants recruited from each health facility (Les Irois, Moron, Jean Baptiste de l’Anse d’Hainault, and Petite Rivière), with facility catchment areas generated by mapping the minimum bounding geography (convex hull) of the household locations for participants recruited at each facility.


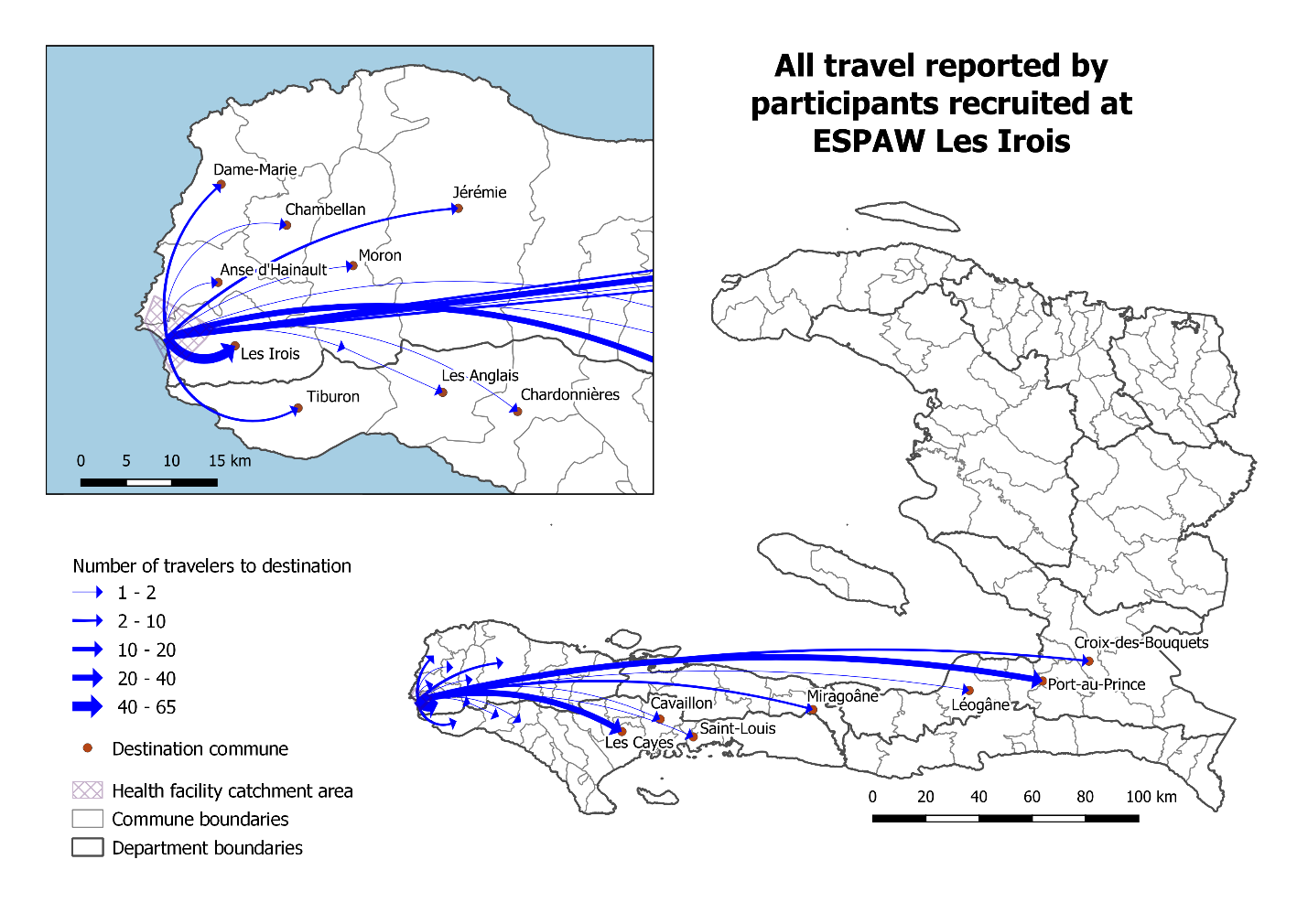


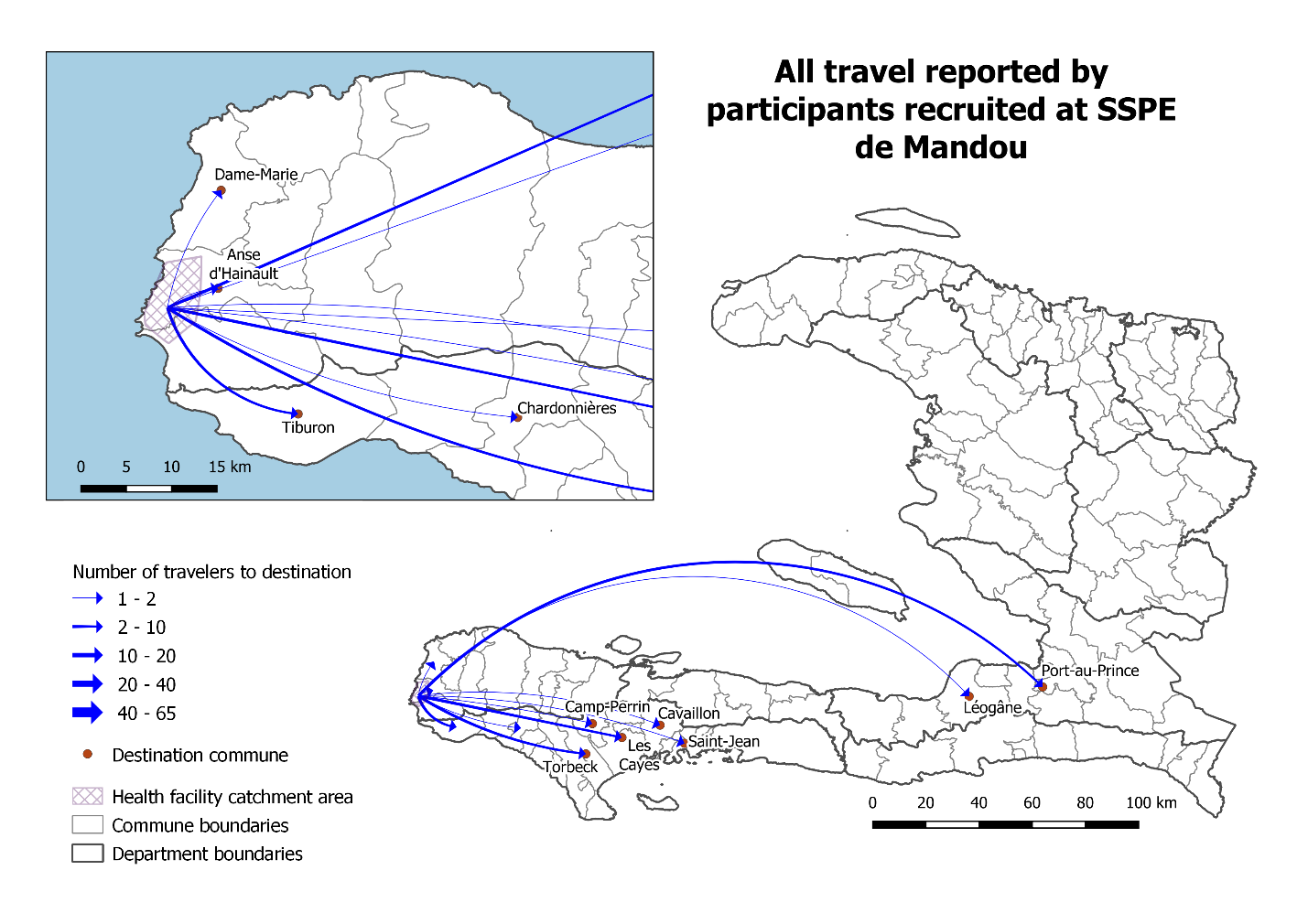


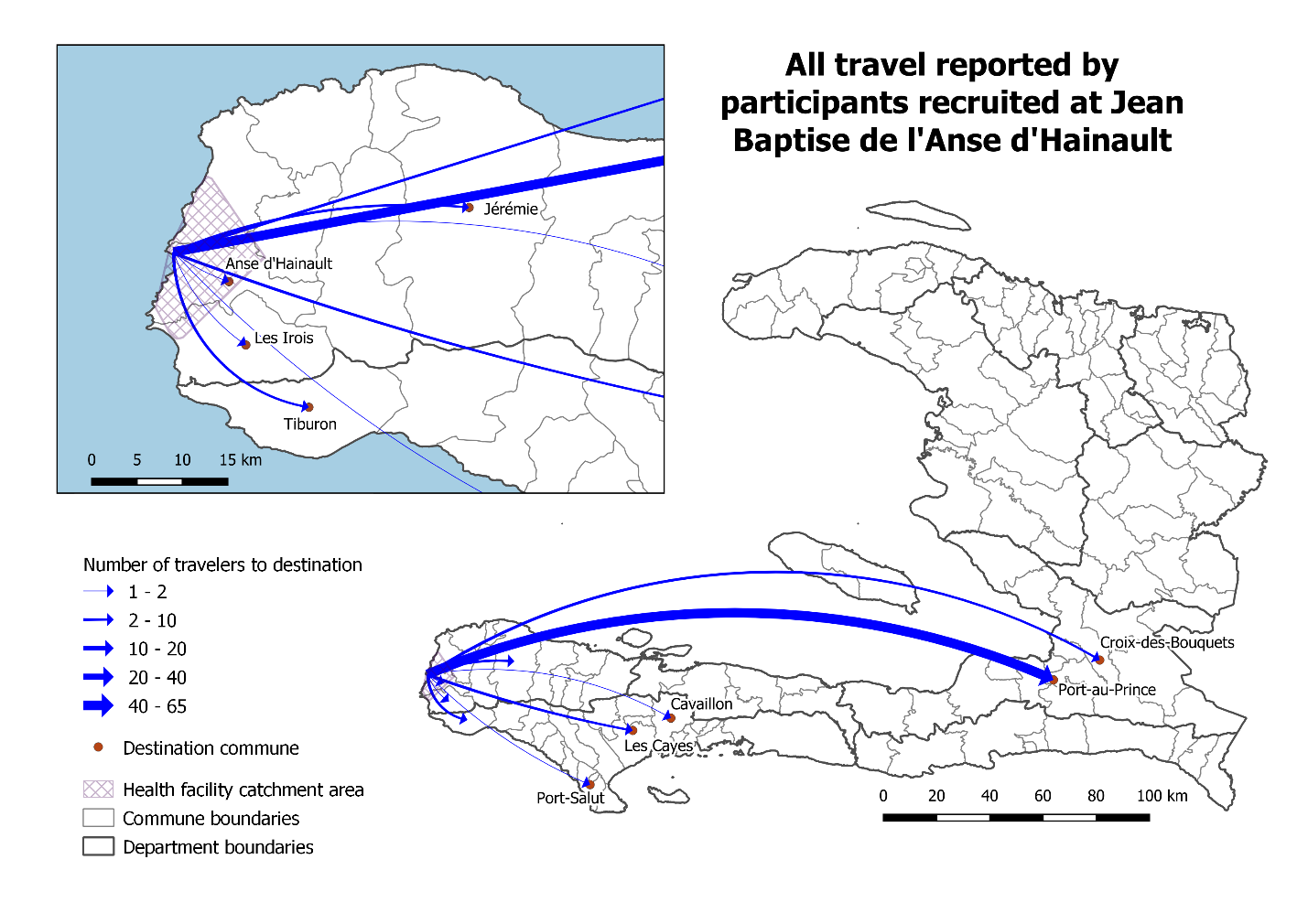


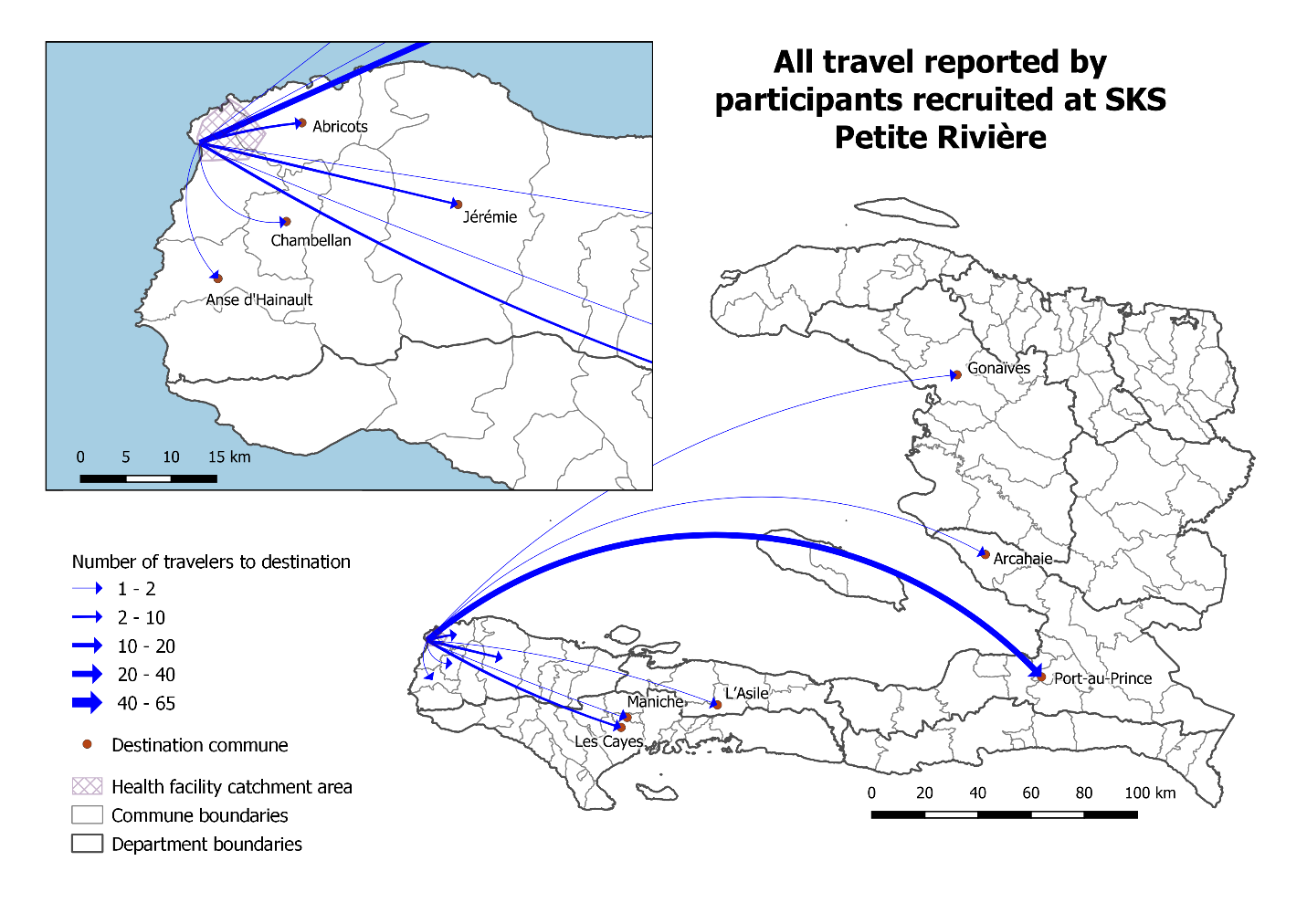

Supplement: Supplementary file 1 [file tpmd200097.SD1.docx]
